# Characterization of Two Neutralizing Antibodies against Rift Valley Fever Virus Gn Protein

**DOI:** 10.3390/v12030259

**Published:** 2020-02-27

**Authors:** Meng Hao, Guanying Zhang, Shengnan Zhang, Zhengshan Chen, Xiangyang Chi, Yunzhu Dong, Pengfei Fan, Yujiao Liu, Yi Chen, Xiaohong Song, Shuling Liu, Changming Yu, Jianmin Li, Xianzhu Xia

**Affiliations:** 1 Comparative Medicine Center, Peking Union Medical College (PUMC) and Institute of Laboratory Animal Sciences, Chinese Academy of Medical Sciences (CAMS), Beijing 100021, China; haorm_23@126.com; 2 Beijing Institute of Biotechnology, Beijing 100071, China; zhangguanying_123@outlook.com (G.Z.); goodnightcxy@163.com (X.C.); 13811429044@163.com (Y.D.); fanpengfei93@163.com (P.F.); yjliu418@outlook.com (Y.L.); chenyi510@icloud.com (Y.C.); m13810676152@163.com (X.S.); lslsjy2203@sina.com (S.L.); 3 College of Wildlife and Protected Areas, Northeast Forestry University, Harbin 150040, China; Zhang_Shengnan1992@163.com; 4 Tianjin Institue of Envionmental and Operational Medicine, Key Laboratory of Risk Assessment and Control for Environment and Food Safety, Tianjin 300050, China; 12350981150805@alumni.sjtu.edu.cn; 5 Changchun Veterinary Research Institute, Chinese Academy of Agricultural Sciences, Changchun 130000, China

**Keywords:** Rift Valley fever virus (RVFV), neutralizing antibody (NAb), Gn protein, critical residues

## Abstract

The Rift Valley fever virus (RVFV) is an arthropod-borne virus that can not only cause severe disease in domestic animals but also in humans. However, the licensed vaccines or available therapeutics for humans do not exist. Here, we report two Gn-specific neutralizing antibodies (NAbs), isolated from a rhesus monkey immunized with recombinant human adenoviruses type 4 expressing Rift Valley fever virus Gn and Gc protein (rHAdV4-GnGcopt). The two NAbs were both able to protect host cells from RVFV infection. The interactions between NAbs and Gn were then characterized to demonstrate that these two NAbs might preclude RVFV glycoprotein rearrangement, hindering the exposure of fusion loops in Gc to endosomal membranes after the virus invades the host cell. The target region for the two NAbs is located in the Gn domain III, implying that Gn is a desired target for developing vaccines and neutralizing antibodies against RVFV.

## 1. Introduction

The Rift Valley fever virus (RVFV), a member of the *Phlebovirus* genus in the order *Bunyavirales*, poses a threat to both human and livestock health. RVFV can be transmitted by mosquito bite or contact with infected animals. The clinical symptoms caused by RVFV infection can differ according to the severity of the infection, including being asymptomatic (in 30%–60% of cases) or causing a weeklong unexplained febrile illness, severe hemorrhagic fever, encephalitis, retinitis, and potentially death [1]. Since this virus was first isolated in 1931, epidemics have been reported on the African continent, such as in Egypt, South Africa, Mauritania, and East Africa [2,3,4,5]. Due to globalization and climate change, Rift Valley fever (RVF) spread to Saudi Arabia and Yemen via infected livestock trade in September 2000, resulting in the deaths of hundreds of people, which was the first reported occurrence of the disease outside the African continent [6,7]. In 2016, the first imported case was reported in China, where the patient returned to China from Angola [8]. These cases indicate that RVFV has the potential for cross-boundary transmission. At present, however, there are no licensed vaccines and antiviral drugs for use in humans [9].

RVFV is a *Phlebovirus* under the family *Phenuiviridae* in the order *Bunyavirales* [10]. RVFV contains a single-stranded, negative-sense tripartite RNA genome consisting of a large (L), medium (M), and small (S) segment [11]. The L segment encodes viral RNA-dependent RNA polymerase. The S segment encodes NSs protein and N protein in an ambisense manner. The viral glycoproteins, Gn and Gc, are encoded by the M segment [11]. The Gn and Gc proteins are important structural proteins on the viral surface which mediate receptor recognition and membrane fusion [12,13]. The Gn and Gc proteins exist as heterodimers on the surface of virus particles, which is similar to another *Phlebovirus* member, the severe fever with thrombocytopenia syndrome virus (SFTSV) [14,15,16]. Several structural studies have indicated that the fusion loop on the Gc protein is shielded by the Gn protein before virus binding to host cells [16,17]. Therefore, the Gn and Gc proteins are the primary targets for developing vaccines and neutralizing antibodies (NAbs). A few decades ago, several studies have shown that there exist virus-neutralizing epitopes on the glycoproteins [18,19,20]. In 2018, Allen et al. isolated some NAbs from rabbits, which were directed to the membrane-distal domain of RVFV Gn and can preclude virus entry into a host cell [21]. In 2019, Wang et al. isolated eight Gn-specific NAbs from a convalescent RVF patient that can block the binding of virions to the host cells [22].

In this study, two NAbs that target Gn proteins were isolated from a rhesus monkey immunized with recombinant human adenoviruses type 4 expressing RVFV Gn and Gc proteins (rHAdV4-GnGcopt). We characterized affinities and neutralizing epitopes of these antibodies and further tried to explain possible neutralizing mechanisms through antibody–antigen molecular docking based on bioinformatics.

## 2. Materials and Methods

### 2.1. Cells, Viruses, and Animals

Huh7 cells were cultured in Dulbecco’s modified Eagle’s medium (DMEM) (Gibco, Grand Island, NY, USA) supplemented with 10% fetal bovine serum (FBS) (Gibco, Grand Island, NY, USA) and maintained at 37 °C with 5% CO_2_. Peripheral blood mononuclear cells (PBMCs) and sera were isolated from a rhesus monkey that was immunized with rHAdV4-GnGcopt after 210 days, and PBMCs were stored in liquid nitrogen for further use. RVFV MP-12 strains expressing eGFP (RVFV-SeGFP) and RVFV MP-12 strains expressing *Renilla* luciferase (RVFV-SRluc) were rescued using a previously described method [23]. The animal experiments in this study were approved by the Animal Welfare and Ethics committee of Academy of Military Medicine (Permit Number E20181030 and Approval Date 30-10-2018). All institutional and national guidelines for the care and use of animals were followed.

### 2.2. Virus Neutralization Test (VNT)

Sera were heat-inactivated for 2 h at 56 °C before use. Threefold serial dilutions of sera (40-fold in the initial dilution) in 96-well plates (CoStar, Washington, DC, USA) were incubated with an equal volume of RVFV-SeGFP (100 TCID_50_ per well) for 1.5 h at 37 °C. Huh7 cells (20,000) were then added to each well, followed by incubation in a 37 °C, 5% CO_2_ incubator for 48 h. The eGFP was detected using Cytation1 (Bio-Tek, Winooski, VT, USA). The neutralizing antibody titers were calculated using the Reed–Muench method [24]. To measure the neutralization activity of each RVFV monoclonal antibody, the isolated monoclonal antibodies were serially diluted in 96-well plates (CoStar, Washington, DC, USA) and incubated with an equal volume of RVFV-SRluc (100 TCID_50_ per well) for 1.5 h at 37 °C. Then, 20,000 Huh7 cells were added to each well. The cells were incubated in a 37 °C, 5% CO_2_ incubator for 24 h. The luciferase activity (Luc) was measured via the *Renilla* luciferase assay system (Promega, Madison, WI, USA). The neutralization of RVFV (%) was calculated as (1 − Luc_measured_/Luc_virus control_) × 100. Sigmoid neutralizing curves were generated through GraphPad Prism 8 (GraphPad, San Diego, CA, USA).

### 2.3. Isolation of Gn-Specific Single Memory B Cells

PBMCs were stained with anti-CD3-PerCP (BD, Franklin Lakes, NJ, USA), anti-CD19-Alexa Flour 700 (Beckman Coulter, Pasadena, CA, USA), anti-Human IgG-PE (BD, Franklin Lakes, NJ, USA), and Strep-tag Gn at 2 μg/5 × 10^5^ cells. After they were washed three times with FPBS (2% FBS), the PBMCs were stained with anti-Strep-APC (Biolegend, San Diego, CA, USA). Gn-specific memory B cells were defined as CD19^+^ CD3^−^ hIgG^+^ Strep^+^ and sorted into 96-well plates (single cell/well) using a MoFlo XDP cell sorter (Beckman Coulter, Pasadena, CA, USA).

### 2.4. Amplification of Antibody Variable Region Genes and Expression of Antibodies from Linear Expression Cassettes

The genes encoding Ig VH and VL chains were amplified by RT-PCR and nested PCR using a previously described method [25]. In summary, the single cells sorted in 96-well plates were served as templates to generate cDNA of Ig VH and VL by RT-PCR using SuperScript™ III First-Strand Synthesis System (Invitrogen, Waltham, MA, USA). The Ig VH and VL genes were produced via two rounds of nested PCR. The cDNAs were regarded as a template to amplify the products of first-round nested PCR. Then, the Ig VH and VL genes were amplified from the first round of nested PCR products. The cytomegalovirus promoter, Ig leader sequence, Ig VH/VL genes, Ig constant region (IgG1), and poly(A) sequence segments were linked by overlapping PCR to generate linear expression cassettes, which include full-length Ig heavy- or light-chain, as previously described [26]. The purified PCR products of paired full-length Ig H and L genes were co-transfected into HEK293T cells using TurboFect transfection reagent (Thermo Scientific, Waltham, MA, USA). The culture supernatants were collected at 48 h after transfection and stored at −20 °C for further use.

### 2.5. Gene Construction

The linear expression cassettes, which include full-length Ig heavy- or light-chain, served as templates to amplify the full-length Ig gene using specific primers. The full-length Ig genes were ligated into the pCDNA3.4 plasmid for monoclonal antibody large-scale expression using EcoRI and BamHI restriction sites using NEBuilder^®^HiFi DNA Assembly Master Mix (NEB, Ipswich, MA, USA).

To express truncated Gn proteins via the prokaryotic expression system, the genes of truncated Gn proteins were cloned into the pET-32a vector. In particular, RVFV Gn (M residues 154–469, GenBank accession number: DQ380208.1) was truncated into 10 segments (45 aa each) and two adjacent segments overlapped by 15 amino acids, named Gn1a–Gn10a. Each segment was individually cloned into the EcoRI and XhoI restriction sites of the pET-32a vector.

### 2.6. Enzyme-Linked Immunosorbent Assay (ELISA)

The RVFV purified truncated Gns (Gn1a–Gn10a) and RVFV Gn were coated at 0.2 μg per well on 96-well plates at 4 °C overnight. The plates were washed three times with PBST (0.2% Tween 20 in PBS) and blocked with 2% BSA at 37 °C for 1 h. The plates were washed three times with PBST, and the threefold serial dilutions of serum samples in PBST containing 0.2% BSA (starting at 1:100 dilution) were added for 1 h at 37 °C. Plates were washed three times with PBST, and an HRP-conjugated anti-human IgG antibody (Abcam, Cambridge, UK) was added for 1 h at 37 °C. Then, 3,3′,5,5′-tetramethylbenzidine substrate (Solarbio, Beijing, China) was added after being washed three times and 2 M H_2_SO_4_ was added to stop the reaction. The optical density (OD) was measured at 450–630 nm (OD_450_–OD_630_).

### 2.7. Biolayer Interferometry (BLI)

The kinetics of antibody binding to RVFV Gn proteins were analyzed using biolayer interferometry (BLI) via a ForteBio Octet instrument (Pall Life Science, Port Washington, NY, USA). Briefly, anti-hIgG Fc Capture Biosensors (Pall Life Science, Port Washington, NY, USA) were loaded with antibodies at 20 μg/mL diluted in PBST containing 0.05% Tween 20. After loading, the sensors were washed with loading buffer for 60 s to remove excess unbound antibodies to regenerate a baseline signal. Then, the sensors were placed in wells containing RVFV Gn at serial concentrations ranging from 0 to 1000 nM in loading buffer to test the (on-rate) association rate. Finally, the sensors were placed in loading buffer wells to test the (off-rate) dissociation rate. Equilibrium dissociation constant (*K_D_*) values were generated from the ratio of off-rate values to on-rate values. Data were analyzed through the ForteBio Data Analysis software 7.0 (Molecular Devices LLC, Fremont, CA, USA).

### 2.8. Protein Expression and Purification

For large-scale antibody expression, pCDNA3.4 plasmids containing genes corresponding to antibody heavy- and light-chains were co-transfected at a 1:1 ratio into Expi293F mammalian suspension cells following the manufacturer’s instructions (Thermo Fisher, Waltham, MA, USA). Antibody supernatants were harvested four days after transfection and purified via protein A affinity chromatography according to the manufacturer’s protocol (GE Healthcare, Chicago, IL, USA).

The Gn1a–Gn10a genes were cloned into pET-32a and transformed into *Escherichia coli* BL21(DE3) competent cells (Tiangen, Beijing, China). The culture was grown at 37 °C in LB medium supplemented with 100 μg/mL of ampicillin until the OD_600_ reached 0.8. The cultures were inducted with the final concentration of 0.5 mM of isopropyl-β-d-thiogalactopyranoside (IPTG). The target proteins were subsequently harvested by lysing *E. coli* via ultrasonic waves and purified using a HisTrap HP column (GE Healthcare, Chicago, IL, USA).

### 2.9. Amino Acid Mapping of Epitopes by Alanine-Scanning Mutagenesis

The polypeptides ^409^GSKKCTGDAAFCSAY^423^, contained in segment Gn9a, were subjected to alanine-scanning mutagenesis (alanine residues were mutated to serine) to generate 15 segments, named Gn9b–Gn9p. Each segment was also individually cloned into the EcoRI and XhoI restriction sites of the pET-32a vector, expressed via a prokaryotic expression system, and purified using a HisTrap HP column (GE Healthcare, Chicago, IL, USA). Then, these purified segments were coated on 96-well plates (2 μg/mL, 100 μL/well) and incubated with 1332F11 (0.5 μg/mL) and 1331E4 (0.01 μg/mL). NAb concentrations were determined using median effective concentration (EC50) against Gn9a to ensure that signals were within the linear range of detection. NAbs were detected using an HRP-conjugated anti-human IgG antibody (Abcam, Cambridge, UK). Antibody reactivity against each mutant segment was calculated relative to wild-type Gn9a reactivity by normalizing to the OD_450_–OD_630_ from wild-type Gn9a. Mutations were considered critical to the NAb epitope if they did not support reactivity of the NAb.

### 2.10. Western Blotting

The RVFV Gn was fractionated via sodium dodecyl sulfate polyacrylamide gel electrophoresis, then electroblotted to polyvinylidene fluoride membranes and incubated with NAb at a final concentration of 1 μg/mL or anti-Gn rabbit polyclonal antibody (1:4000 dilution). The corresponding secondary antibodies labeled with HRP were used at a final concentration of 0.1 μg/mL. The Millipore chemiluminescent substrate (Merck Millipore, Burlington, MA, USA) was added for detecting RVFV Gn according to the manufacturer’s instructions.

### 2.11. Antigen–Antibody Molecular Docking

The atomic structures of the variable region of NAbs were initially modeled based on their amino acid sequences via Discovery Studio 4.5 (BIOVIA, San Diego, CA, USA). Then, the computational antigen–antibody molecular docking program ZDOCK, integrated in Discovery Studio 4.5, was used to generate a simulation conformation model for NAbs to RVFV Gn, of which the atomic structure was obtained from the PDB database (PDB ID: 5Y0W).

## 3. Results

### 3.1. Sera Response in a Rhesus Monkey Immunized with rHAdV4-GnGcopt

**Figure 1:**
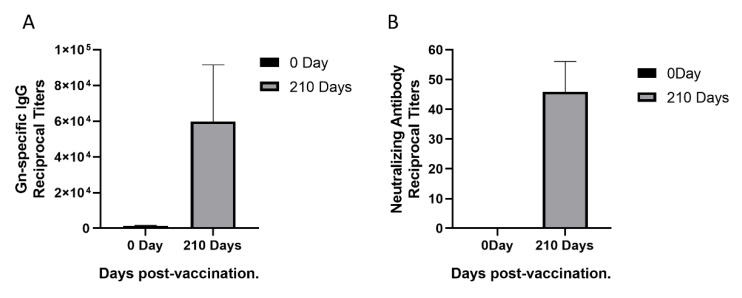
Sera response in a rhesus monkey immunized with recombinant human adenoviruses type 4 expressing Rift Valley fever virus Gn and Gc protein (rHAdV4-GnGcopt). (**A**) Serum samples were collected at 0 and 210 days after immunization and subjected to ELISA analysis of IgG antibodies to the Rift Valley fever virus (RVFV) Gn. The titers were calculated as reciprocal endpoints. The cutoff values were calculated as the mean optical density of control serum (1:8100 dilution) multiplied by 2.1. Data are presented as the means ± standard deviations (error bars) from three independent trials. (**B**) Serum samples were collected at 0 and 210 days after immunization and subjected to virus neutralization test via RVFV MP-12 strains expressing eGFP (RVFV-SeGFP). The neutralizing antibody titers were calculated using the Reed–Muench method. Data are presented as the means ± standard deviations (error bars) from three independent trials.

To isolate monoclonal antibodies from a female rhesus monkey immunized with rHAdV4-GnGcopt after 210 days, we first performed ELISA to measure Gn-specific IgG titers in the rhesus monkey. The Gn-specific IgG titers increased remarkably (Figure 1A). To this end, we also measured neutralizing antibody titers in the monkey using VNT. Compared with neutralizing antibody titers in the sera collected pre-vaccination, neutralizing antibody titers in the sera collected 210 days post-vaccination increased obviously (Figure 1B). Taken together, the results indicated that a good level of Gn-specific and neutralizing antibody responses were effectively induced by the rHAdV4-GnGcopt.

### 3.2. Isolation and Binding Characterization of Gn-Specific Neutralizing Antibodies

**Figure 2:**
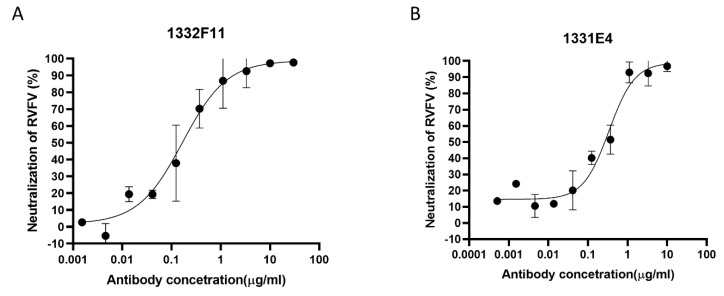
Protection efficacy of monoclonal antibodies against RVFV infection in Huh7 cells. (**A**,**B**) RVFV MP-12 strains expressing *Renilla* luciferase (RVFV-SRluc) amplified in Huh7 cells were mixed with threefold serial dilutions of the indicated neutralizing antibodies (NAbs). The luciferase activity (Luc) was measured via the *Renilla* luciferase assay system. The neutralization of RVFV (%) was calculated as (1 − Luc_measured_/Luc_virus control_) × 100. Sigmoid neutralizing curves were generated through GraphPad Prism 8. Experiments were repeated three times, and the representative results are presented.

Based on the results described above, Gn-specific memory B cells defined as CD19^+^ CD3^−^ hIgG^+^ Strep^+^ were sorted from PBMCs as single cells through fluorescent cell sorting (Supplementary Figure S1). Fifty-seven pairs of VH-Vκ genes and 47 pairs VH-Vλ genes were amplified from Gn-specific memory B cells. To rapidly express and screen Gn-specific antibody genes, linear expression cassettes containing either the full-length Ig heavy- or light-chain were co-transfected into a HEK293T cell expression system in 96-well plates. Then eight κ subtype antibodies and seven λ subtype antibodies, targeting Gn, were screened from the transfected culture supernatants via ELISA (Supplementary Tables S1 and S2). In order to study neutralization activities and the binding characterization of these positive clones, the full-length Ig heavy- or light-chain genes of these clones were cloned into plasmid pCDNA3.4, expressed at large scale using an Expi293F cell expression system, and purified using protein A affinity chromatography. Then RVFV-SRluc was applied to determine whether these 15 purified antibodies could prevent the virus from infecting Huh7 cells. Finally, two NAbs that were screened from these positive clones using VNT exhibited good neutralizing capabilities, of which the median inhibitory concentrations (IC_50_) were 0.1678 and 0.3388 μg/mL (Figure 2). To identify variable region gene segments and somatic hypermutation (SHM), the sequences of two NAbs were then analyzed using IMGT/V-QUEST (www.imgt.org/IMGT_vquest/input). The genes of the heavy-chain variable regions of two NAbs were found to originate from two different germline genes (HV4-2*01 and HV3-18*01), and the light-chain variable regions genes of the two NAbs originated from two different germline genes (KV2S8*01 and LV6-5*01). Two NAbs showed low SHM rates compared with their germline genes, with nucleotide identities ranging from 89.58% to 96.23% for the variable regions in both the heavy- and light-chains (Supplementary Table S3). In addition, the VL genes of the two NAbs differed: 1332F11 used a κ chain while 1331E4 used λ chain.

To ascertain the binding affinities of two NAbs, we tested the kinetics of NAb binding to RVFV Gn through BLI. In this assay, the binding affinity (*K_D_*) of two NAbs was measured by the ratio of off-rate values (*k*_dis_) to on-rate values (*k*_on_). Compared with 1332F11 (*k*_on_ = 7.78 × 10^4^ M^−1^s^−1^), 1331E4 (*k*_on_ = 4.09 × 10^5^ M^−1^s^−1^) could bind with Gn more easily. Moreover, 1331E4 (*k*_dis_ = 3.84 × 10^−4^s^−1^) is more difficult to dissociate from Gn than 1332F11 (*k*_dis_ = 1.25 × 10^−3^s^−1^).Therefore, the results demonstrated that the binding affinity of 1331E4 (*K_D_* = 0.938 nM) to RVFV Gn was 17 times higher than that of 1332F11 (*K_D_* = 16 nM)to RVFV Gn, even though the protective efficiency of two NAbs in Huh7 cells was approached (Table 1).

### 3.3. Identification of the Binding Epitopes of Two NAbs

**Figure 3:**
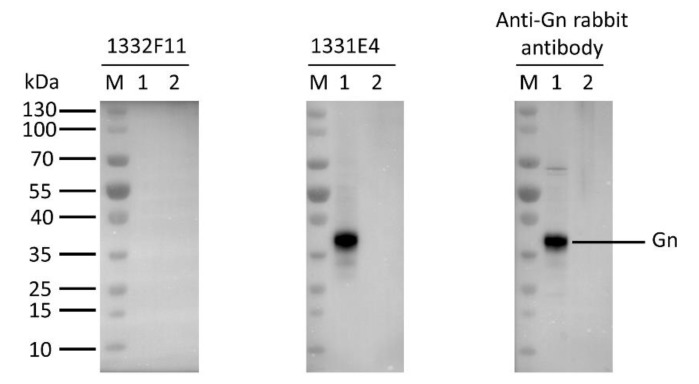
Western blot analysis of the type of NAb epitopes. The supernatants, collected from the Expi293F cells transfected with Gn expression vector (pCDNA3.4-Gn) and empty vector (pCDNA3.4-empty), were loaded into lane 1 and lane2, respectively. The indicated antibodies were incubated with the membrane transferred with RVFV Gn at a final concentration of 1 μg/mL. The protein reacting with anti-Gn rabbit polyclonal antibody was used as a positive control.

**Figure 4:**
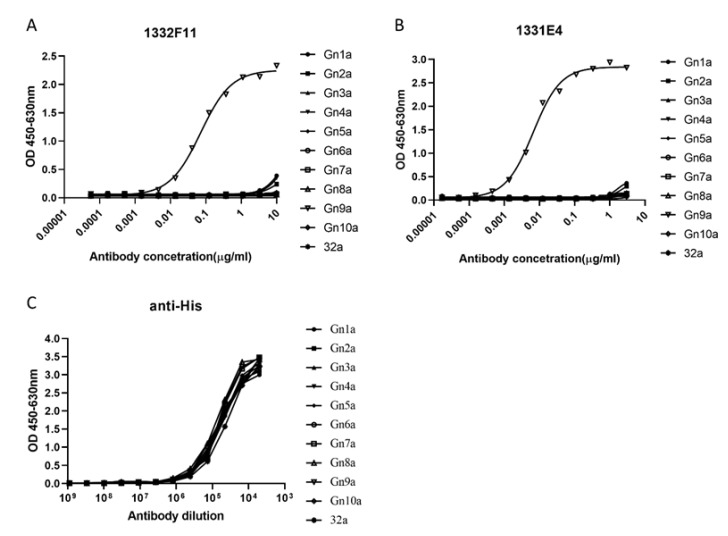
ELISA analysis of NAbs to Gn truncated segments. (**A**,**B**) The truncated segments, coated at 0.2 μg/well in a 96-well plate, were incubated with threefold serial dilutions of the indicated NAbs. 1332F11 and 1331E4 were diluted at 10 μg/mL and 3 μg/mL in an initial well, respectively. 32a represents pET-32a empty vector. (**C**) The anti-His antibody (1:5000 initial dilution) was used as positive control. The optical density (OD) was measured at OD_450_–OD_630_. Sigmoid curves were generated through GraphPad Prism 8. Sigmoid neutralizing curves were generated through GraphPad Prism 8.

**Figure 5:**
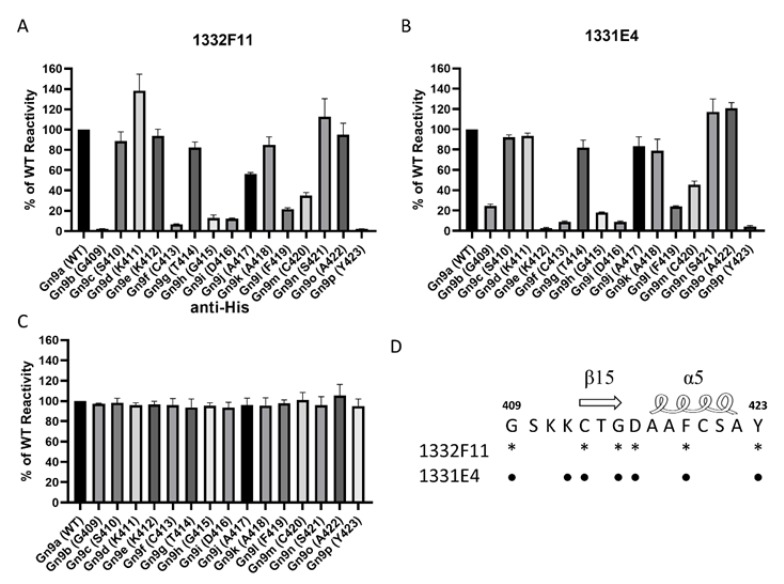
Mapping of NAb epitopes based on the polypeptide ^409^GSKKCTGDAAFCSAY^423^ in Gn9a using alanine-scanning mutagenesis. (**A**,**B**) These purified segments, coated on a 96-well plate (0.2 μg/well), were incubated with 1332F11 (0.5 μg/mL) and 1331E4 (0.01 μg/mL). Antibody reactivity against each mutant segment was calculated relative to wild-type (WT) Gn9a reactivity by normalizing to the OD_450_–OD_630_ from wild-type Gn9a. (**C**) The anti-His antibody (1:5000 initial dilution) was used as positive control. Data are presented as the means ± standard deviations (error bars) from three independent trials. (**D**) The critical residues for 1332F11 are labeled with asterisks, and the critical residues for 1331E4 are labeled with black dots.

Western blotting was used to study whether the two NAbs could recognize RVFV Gn under reducing conditions. The results show that 1332F11 could recognize RVFV Gn in reduced conditions, but 1331E4 failed to recognize RVFV Gn under the same conditions (Figure 3). To further determine the epitopes on RVFV Gn targeted by the two NAbs, we truncated Gn into 10 segments (Table 2) and measured the binding capacity between these segments and NAbs using ELISA. Interestingly, the results revealed that the two NAbs could both recognize segment Gn9a (Figure 4). Furthermore, the two NAbs could not bind either segment Gn8a or Gn10a, despite the fact that each contains 15 amino acid residues that overlap with Gn9a. Hence, we speculated that polypeptides ^409^GSKKCTGDAAFCSAY^423^ (residues number 409–423), contained in Gn9a, were likely the target peptide for the two NAbs. In a previous study, Wu et al. demonstrated that RVFV Gn consists of three subdomains (domains I, II, and III) and there is one β-sheet and one α- helix existing in polypeptides ^409^GSKKCTGDAAFCSAY^423^ in domain III (Figure 5D) [16].

To further confirm the residues in Gn9a critical for binding the two NAbs, we mapped these amino acid residues using alanine-scanning mutagenesis. Each amino acid residue in the polypeptides ^409^GSKKCTGDAAFCSAY^423^, carried by Gn9a, was individually mutated to an alanine (alanine residues were mutated to serine) (Table 3). The results show that six amino acids (G409, C413, G415, D416, F419, and Y423) within Gn9a were critical for recognition of Gn by 1332F11, and seven amino acids (G409, K412, C413, G415, D416, F419, and Y423) were identified as critical in the case of 1331E4 (Figure 5A,B). Interestingly, even though 1331E4 and 1332F11 are two distinct antibodies, the six amino acids critical for binding and recognition by 1332F11 were also critical for 1331E4.

### 3.4. Modeling the Interaction of NAbs and Gn

**Figure 6:**
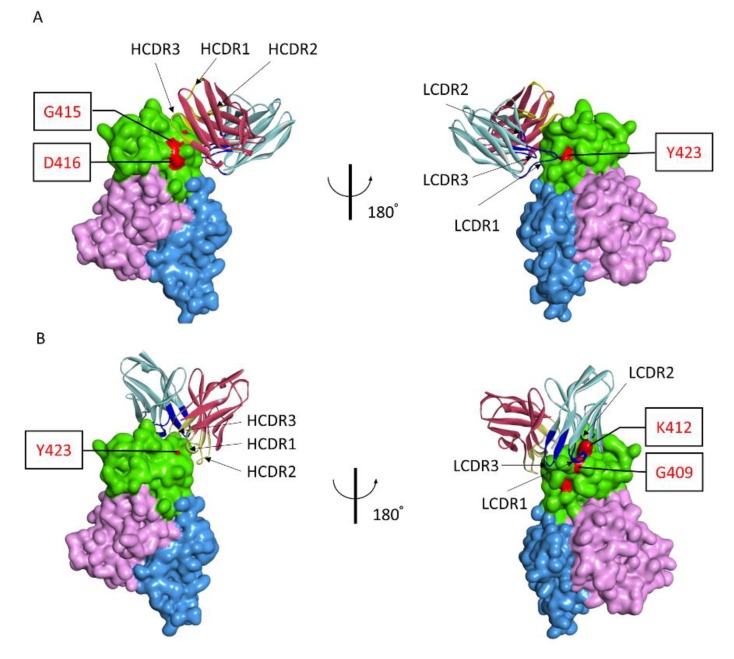
Antigen–antibody molecular docking of two NAbs with RVFV Gn. (**A**) Overall structure of Gn and 1332F11 complex. Domain III of Gn is presented as green, domain I is presented as pink, and domain II is presented as blue. The red regions in domain III represent critical residues for 1332F11. For 1332F11, dark red indicates the heavy-chain, and light-blue represents the light-chain. CDRs in heavy-chain and light-chain are shown in yellow and dark blue, respectively. (**B**) Overall structure of Gn and 1331E4 complex, as presented in (**A**).

Based on the residues in polypeptide ^409^GSKKCTGDAAFCSAY^423^ that were identified as being critical for recognition by 1332F11 and 1331E4, we used the ZDOCK program to gain additional insights into the molecular basis of NAb binding with RVFV Gn and to determine the possible neutralizing mechanism. Then, the atomic structures of the variable region of NAbs and Gn docking were conducted to generate 2000 possible binding poses, with a ranking based on comprehensive evaluations of computational docking scores. According to the docking scores and experimental mutagenesis data, the most reasonable poses of two NAbs were selected. Computational docking suggested that the two NAbs bound to domain III in Gn in different orientations, even though the epitopes were very similar (Figure 6). 1332F11 bound to the region near domain II in Gn. The complementarity determining region (CDR) loops 3 (HCDR3) of the heavy-chain were used to bind a region constituted by residues G415 and D416. Meanwhile, the light-chain bound to residue Y423 through LCDR1 (Figure 6A). Thus, a trough in 1332F11, formed by HCDR3 and LCDR1, was penetrated by one β-sheet and one α-helix in domain III of Gn. Differently from 1332F11, 1331E4 bound to the apex of domain III in Gn (Figure 6B). The heavy-chain used its HCDR3 to bind to residue Y423, and the light-chain used its two CDR loops (LCDR1 and LCDR2) to bind to residues G409 and K412, respectively. In this interaction, the key residues G409, K412, and Y423 in Gn provided a platform for binding 1331E4.

**Figure 7:**
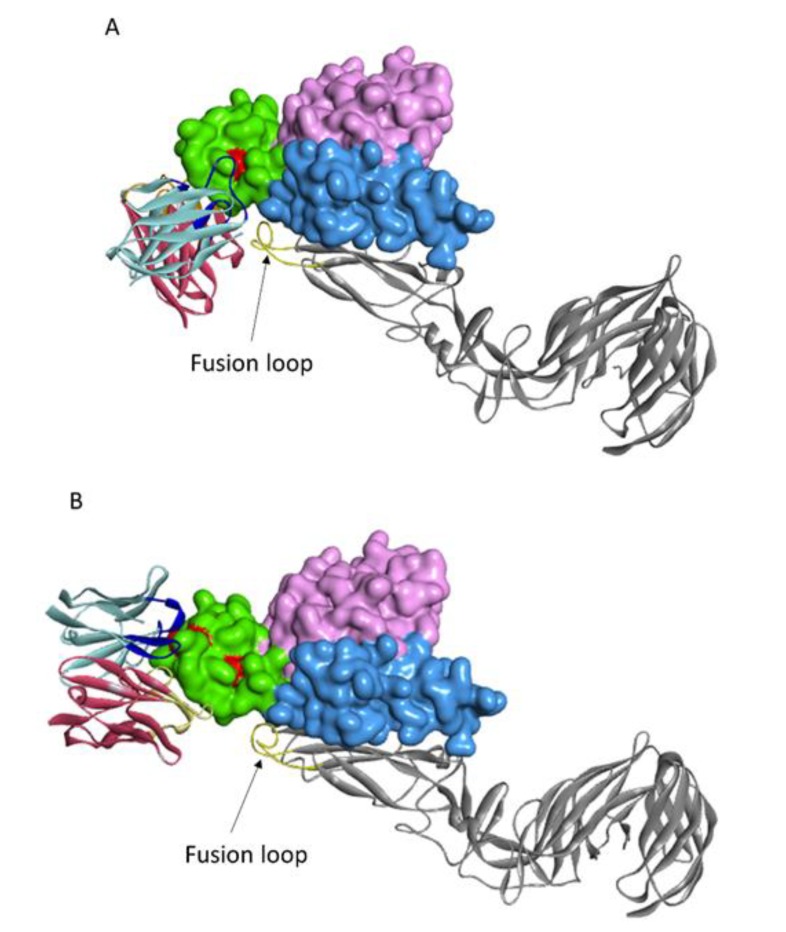
Modeling structure of NAbs and RVFV Gn–Gc assembly. (**A**) Overall structure of 1332F11 and RVFV Gn–Gc assembly. Fusion loop in Gc is shown as gold, and the rest of Gc is indicated as silver gray. NAbs and Gn are described as in Figure 6. (**B**) Overall structure of 1331E4 and RVFV Gn–Gc assembly, as presented in (**A**).

As previously described, Gn and Gc proteins exist as a heterodimer on the viral surface [15,27]. Next, we superimposed the Gn component of our NAb–Gn docking model onto a reported model of assembled RVFV Gn–Gc (PDB ID: 6F9F). The results show that the sites in Gn that supporting binding of NAbs were close to the fusion loop in Gc (Figure 7).

## 4. Discussion

In this study, we isolated two RVFV-neutralizing antibodies from a rhesus monkey immunized with rHAdV4 expressing RVFV Gn and Gc proteins and characterized the key residues for two NAbs. Before isolating a neutralizing antibody to Gn from this monkey, we primarily tested neutralizing antibody titers in sera using RVFV MP-12 strains expressing eGFP, which can be worked with at Biosafety Level 2 [28]. Furthermore, the neutralizing antibody titers are high enough to apply Gn-specific memory B cell sorting and single-cell PCR technology to screen antibodies against Gn [29,30]. We then isolated two NAbs against Gn from this monkey and measured the IC_50_ of two NAbs through RVFV MP-12 strains expressing *Renilla* luciferase in a Biosafety Level 2 laboratory, based on a method modified from previously described luciferase-reporter bioassay with Huh7 cells [31]. However, more experiments are needed to confirm the comparability of this method with the traditional plaque reduction neutralization test. The two NAbs showed good neutralizing activities, precluding RVFV infection with IC_50_ values of 0.1678 and 0.3388 μg/mL. One of two NAbs uses a κ light-chain and another one incorporates a λ light-chain. Despite the different types of light-chains incorporated by the two NAbs, the germline analysis indicates that both NAbs have relatively low SHM rates. Consequently, the two NAbs demonstrate relatively low binding affinity to Gn. The low SHM rates might be related to the long time after rhesus monkey immunization. Due to RVFV MP-12 being an attenuated virus [32], it is safe to many RVFV-susceptible animals, such as hamsters, cattle, sheep, and pregnant ewes [33,34,35]. Therefore, the virus we rescued could not provide us a good method to evaluate the protection efficiency of NAbs in vivo. Further studies are needed to characterize the inhibitory potential of NAbs in vivo.

Western blotting analysis showed that 1332F11 could not recognize Gn under reducing conditions, but 1331E4 could bind to Gn under the same conditions. This implied that 1332F11 could recognize a conformational epitope in Gn, and that 1331E4 could bind to linear epitope in Gn. Interestingly, a subsequent binding epitope identification assay revealed that two NAbs bind to the same polypeptide, ^409^GSKKCTGDAAFCSAY^423^, in Gn. A previous study presenting the crystal structure of Gn showed that this polypeptide contains one β-sheet and one α-helix [16]. Thus, the β-sheet and α-helix structure could be abolished under reducing conditions, leading to the failure of 1332F11 to recognize Gn under these conditions. Coincidentally, the targeting region of an RVFV-neutralizing antibody (RV-Gn1) has also been structurally mapped and found to be near the polypeptides ^409^GSKKCTGDAAFCSAY^423^ in Gn [21]. In addition, this corresponding region of SFTSV Gn is also targeted by the neutralizing antibody Mab4-5 [16]. Taken together, these results demonstrate that domain III is likely an immunodominant part in Gn among phleboviruses. Therefore, this domain in Gn might be treated as a target for developing antibodies and vaccines. Further alanine-scanning mutagenesis of ^409^GSKKCTGDAAFCSAY^423^ suggested that 1331E4 and 1332F11 share six of the same critical residues (Figure 5), but that K412 is only essential for 1331E4. More studies are still needed to confirm whether the other residues in Gn9a play a role in the interaction between NAbs and Gn.

Computational docking analysis indicated that two NAbs bound to Gn in different orientations, even though they shared similar key residues in domain III in Gn. This might be related to the two NAbs possessing different heavy- and light-chain CDR loops. 1332F11 bound to the region nearby domain II through HCDR3 and LCDR1, forming a trough penetrated by one β-sheet and one α-helix in ^409^GSKKCTGDAAFCSAY^423^.

However, 1331E4 bound to the apex of domain III via HCDR3, with LCDR1 and LCDR2 interacting with a platform formed by key residues G409, K412, and Y423 in Gn. Among these key residues for the two NAbs, Y423 is unique in supporting Gn binding to the two NAbs. Y423 contacts with 1332F11 LCDR1 via phenolic hydroxyl groups, and contacts with 1331E4 HCDR3 via an oxygen ion. Computational antigen–antibody docking, conducted under the guidance of alanine-scanning mutagenesis data, indicated that all key residues of the antigen should theoretically contact with the antibodies. In fact, our NAb–Gn docking model revealed that only three of the six key residues contribute to 1332F11 binding to Gn, and only three of the seven key residues support recognition of Gn by 1331E4. Numerous factors may contribute to this phenomenon. (1) Some amino acid residues may play a role in the interaction between NAbs and Gn through maintaining Gn conformation, for example, F419 (located in the interior of Gn) and C413 forming a disulfide bond with C402 [16]. (2) Computational antigen–antibody docking is a method, based on the bioinformatics of protein interaction, for simulating the natural process of an antibody binding to an antigen, so it has some limitations regarding visualization of the interaction between antibody and antigen when compared with a crystalline structure. Therefore, further studies are still needed to describe the particular interaction between Gn and NAbs, including identification of the key residues in NAbs to Gn.

To explain the possible neutralizing mechanism for 1332F11 and 1331E4, we superimposed the Gn component of our NAb–Gn docking model onto a reported model of assembled RVFV Gn–Gc [17]. Furthermore, the regions in Gn targeted by the two NAbs are near the fusion loop in Gc. Given these observations, it is speculated that 1332F11 and 1331E4 likely preclude the sterical rearrangement RVFV glycoprotein, hindering the exposure of fusion loops in Gc to endosomal membranes after the virus invades a host cell. The neutralizing antibody RV-Gn1, targeting a similar region as 1332F11 and 1331E4, is speculated to exert neutralizing activities through the same mechanism [21].

In conclusion, we isolated two Gn-specific neutralizing antibodies from a rhesus monkey immunized with rHAdV4 expressing Gn and Gc proteins. The two NAbs could protect host cells from RVFV infection. Moreover, the description of interaction between Gn and NAbs revealed that RVFV Gn might be an ideal target protein for developing vaccines and neutralizing antibodies against RVFV.

## Figures and Tables

**Table 1:** Kinetics of NAb binding to RVFV Gn–Gc.

Antibody	*K_D_* (M)	*k*_on_ (1/Ms)	*k*_on_ Error	*k*_dis_ (1/s)	*k*_dis_ Error	Full R^2^
1332F11	1.60 × 10^−8^	7.78 × 10^4^	1.69 × 10^3^	1.25 × 10^−3^	1.29 × 10^−5^	0.991524
1331E4	9.38 × 10^−10^	4.09 × 10^5^	7.55 × 10^3^	3.84 × 10^−4^	1.77 × 10^−4^	0.993053

The *k*_on_, *k*_dis_, and *K_D_* for indicated antibodies were determined by the Fortebio Octet system. Equilibrium dissociation constant (*K_D_*) values were generated from the ratio of *k*_dis_ to *k*_on_. The *k*_on_ represents association rate constant. The *k*_dis_ represents dissociation rate constant.

**Table 2:** The amino acid sequences of truncated Gn peptides.

Segment	Amino Acid Sequence
Gn1a	EDPHLRNRPGKGHNYIDGMTQEDATCKPVTYAGACSSFDVLLEKG
Gn2a	YAGACSSFDVLLEKGKFPLFQSYAHHRTLLEAVHDTIIAKADPPS
Gn3a	EAVHDTIIAKADPPSCDLLSAHGNPCMKEKLVMKTHCPNDYQSAH
Gn4a	LVMKTHCPNDYQSAHHLNNDGKMASVKCPPKYELTEDCNFCRQMT
Gn5a	KYELTEDCNFCRQMTGASLKKGSYPLQDLFCQSSEDDGSKLKTKM
Gn6a	CQSSEDDGSKLKTKMKGVCEVGVQALKKCDGQLSTAHEVVPFAVF
Gn7a	GQLSTAHEVVPFAVFKNSKKVYLDKLDLKTEENLLPDSFVCFEHK
Gn8a	EENLLPDSFVCFEHKGQYKGTMDSGQTKRELKSFDISQCPKIGGH
Gn9a	LKSFDISQCPKIGGH GSKKCTGDAAFCSAY ECTAQYANAYCSHAN
Gn10a	ECTAQYANAYCSHANGSGIVQIQVSGVWKKPLCVGYERVVVKRELS

RVFV Gn (154–469 aa) was truncated into 10 segments (45 aa each) with a 15 aa overlap between two adjacent segments, named Gn1a–Gn10a. The 15 overlapping amino acid residues are highlighted in grey. The polypeptide in Gn9a that was recognized by the two NAbs is highlighted in yellow.

**Table 3:** The peptide sequences used for alanine-scanning mutagenesis of Gn9a.

Peptide	Amino Acid Sequence
Gn9a	LKSFDISQCPKIGGHGSKKCTGDAAFCSAYECTAQYANAYCSHAN
Gn9b	LKSFDISQCPKIGGHASKKCTGDAAFCSAYECTAQYANAYCSHAN
Gn9c	LKSFDISQCPKIGGHGAKKCTGDAAFCSAYECTAQYANAYCSHAN
Gn9d	LKSFDISQCPKIGGHGSAKCTGDAAFCSAYECTAQYANAYCSHAN
Gn9e	LKSFDISQCPKIGGHGSKACTGDAAFCSAYECTAQYANAYCSHAN
Gn9f	LKSFDISQCPKIGGHGSKKATGDAAFCSAYECTAQYANAYCSHAN
Gn9g	LKSFDISQCPKIGGHGSKKCAGDAAFCSAYECTAQYANAYCSHAN
Gn9h	LKSFDISQCPKIGGHGSKKCTADAAFCSAYECTAQYANAYCSHAN
Gn9i	LKSFDISQCPKIGGHGSKKCTGAAAFCSAYECTAQYANAYCSHAN
Gn9j	LKSFDISQCPKIGGHGSKKCTGDSAFCSAYECTAQYANAYCSHAN
Gn9k	LKSFDISQCPKIGGHGSKKCTGDASFCSAYECTAQYANAYCSHAN
Gn9l	LKSFDISQCPKIGGHGSKKCTGDAAACSAYECTAQYANAYCSHAN
Gn9m	LKSFDISQCPKIGGHGSKKCTGDAAFASAYECTAQYANAYCSHAN
Gn9n	LKSFDISQCPKIGGHGSKKCTGDAAFCAAYECTAQYANAYCSHAN
Gn9o	LKSFDISQCPKIGGHGSKKCTGDAAFCSSYECTAQYANAYCSHAN
Gn9p	LKSFDISQCPKIGGHGSKKCTGDAAFCSAAECTAQYANAYCSHAN

The polypeptide ^409^GSKKCTGDAAFCSAY^423^ in Gn9a was subjected to alanine-scanning mutagenesis (alanine residues were mutated to serine) to generate 15 segments, named Gn9b–Gn9p. The rest part of Gn9a is highlighted in grey. The mutant residues are highlighted in red.

## Supplementary Materials

The following are available online at https://www.mdpi.com/1999-4915/12/3/259/s1, Figure S1: Gating strategy for the collection of antigen-specific memory B cells, Table S1: ELISA analysis of binding activity between Gn and κ-type antibodies expressed by HEK293T cells in 96-well plates, Table S2: ELISA analysis of binding activity between Gn and λ-type antibodies expressed by HEK293T cells in 96-well plates, Table S3: Germline analysis of isolated NAbs to RVFV Gn.
