# Supplementary material for: Characterization of Two Neutralizing Antibodies against Rift Valley Fever Virus Gn Protein
Source: Viruses. 2020 Feb 27;12(3):259. doi: 10.3390/v12030259 (PMC7150882; doi:10.3390/v12030259)
Supplement: Supplementary file 1 [file viruses-12-00259-s001.pdf]

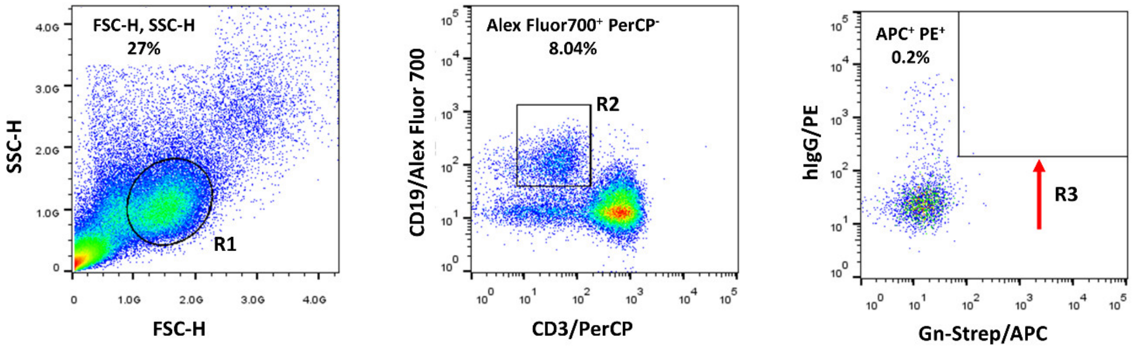

Supplementary Figure 1. Gating strategy for the collection of antigen-specific memory B cells. Isolated PBMCs were incubated with the indicated antibodies. Then, the cells were sorted using a MoFlo XDP cell sorter. Cells were first gated based on the FSC-H and SSC-H (R1). Single cells in R1 were further analyzed via marker expression. In this regard, cells showing expression of CD19, but not CD3 were gated out (R2). Finally, cells in R3, showing hIgG and Gn-specific IgG, were antigen-specific memory B cells, and were collected for further analysis. The percentage displayed in each box indicated the proportion of the boxed cells in the last level of parental cells.

Supplementary Table 1. ELISA analysis of binding activity between Gn and κ-type antibodies expressed by HEK293T cells in 96-well plates

NO.	Clones	OD 450-630nm	NO.	Clones	OD 450-630nm
1	1331A5	0.0631	30	1332A8	0.6584
2	1331A8	0.06	31	1332A9	2.4028
3	1331A9	0.0455	32	1332B1	0.0352
4	1331A10	0.0471	33	1332B2	1.8322
5	1331B1	0.0471	34	1332B6	0.036
6	1331B8	0.0469	35	1332B8	0.035
7	1331B9	0.0494	36	1332B9	0.9046
8	1331C1	0.0492	37	1332B10	0.0542
9	1331C2	0.0472	38	1332B12	0.0364
10	1331C3	0.0477	39	1332C2	0.0313
11	1331D1	0.0542	40	1332C3	0.0281
12	1331D3	0.063	41	1332C7	0.0328
13	1331D7	0.0516	42	1332D7	0.0305
14	1331D10	1.4452	43	1332D8	0.036
15	1331D12	0.0515	44	1332D11	0.0315
16	1331E1	0.0303	45	1332E4	0.033
17	1331E5	1.6005	46	1332E5	0.033
18	1331E6	0.0368	47	1332E10	0.035
19	1331F5	0.0354	48	1332F3	0.051
20	1331F9	0.0345	49	1332F7	0.0593
21	1331F10	0.036	50	1332F8	0.0351
22	1331F12	0.0372	51	1332F11	1.752
23	1331G1	0.0393	52	1332G8	0.0328
24	1331G3	0.0489	53	1332G12	0.0275
25	1331G7	1.2214	54	1332H4	0.0304
26	1331G8	0.0356	55	1332H5	0.0324
27	1331H8	0.0314	56	1332H6	0.0305
28	1331H10	0.0321	57	1332H11	0.0348
29	1332A1	0.0305			

The supernatants were collected from HEK293T cells co-transfected by linear expression cassettes containing either the full-length Ig heavy- or light-chain to screen the positive clones binding to Gn through ELISA. The positive clones (OD 450-630nm > 0.3) were highlighted with red.

Supplementary Table 2. ELISA analysis of binding activity between Gn and λ-type antibodies expressed by HEK293T cells in 96-well plates

NO.	Clones	OD 450-630nm	NO.	Clones	OD 450-630nm
1	1331A3	0.1336	25	1331F10	0.064
2	1331A5	0.0511	26	1331F11	1.8082
3	1331A7	0.0741	27	1331F12	0.0336
4	1331A8	0.0293	28	1331G1	0.0303
5	1331B1	0.0374	29	1331G3	0.0296
6	1331B4	0.0381	30	1331G8	0.0322
7	1331B5	0.0447	31	1331H9	0.0317
8	1331B9	0.0416	32	1331H10	0.0329
9	1331B10	0.7377	33	1332A3	0.3167
10	1331B12	2.1277	34	1332A9	0.0571
11	1331C12	0.0485	35	1332A10	0.0346
12	1331D1	0.0653	36	1332A11	1.0391
13	1331D2	0.0607	37	1332A12	0.0612
14	1331D7	0.0422	38	1332B4	0.648
15	1331D11	0.0294	39	1332B9	0.0308
16	1331E1	0.0333	40	1332D7	0.0291
17	1331E4	0.4697	41	1332E10	0.0295
18	1331E6	0.0365	42	1332F2	0.0245
19	1331E8	0.0331	43	1332F9	0.0303
20	1331E9	0.0366	44	1332G1	0.0276
21	1331E12	0.0322	45	1332G11	0.0267
22	1331F2	0.0558	46	1332H4	0.0443
23	1331F3	0.0391	47	1332H6	0.0357
24	1331F9	0.0499			

The supernatants were collected from HEK293T cells co-transfected by linear expression cassettes containing either the full-length Ig heavy- or light-chain to screen the positive clones binding to Gn through ELISA. The positive clones (OD 450-630nm > 0.3) were highlighted with red.

Supplementary Table 3. Germline analysis of isolated NAbs to RVFV Gn

**Antibody**	**V-H**	**D-H**	**J-H**	**Identity (V-H)**	**CDR3-H**	**V-L**	**J-L**	**Identity (V-L)**	**CDR3-L**	**SHM (H, L)**
1332F11	HV4-2*01	HD1-7*01	HJ5-1*01	89.58%	ARGPGDV	KV2S8*01	KJ1*01	93.88%	MQGTLLPWT	20, 9
1331E4	HV3-18*01	HD6-1*01	HJ5-2*02	92.86%	APGGVGTRA	LV6-5*01	LJ2*01	96.23%	QSYDNSDYWI	11, 8

The *rhesus monkey* germline genes matching the V, D, and J of the heavy-chain (V-H, D-H, and J-H), and V and J of the light-chain (V-L and J-L) from the indicated antibodies. Nucleotide sequence identities with the best-matched V-H and V-L genes are also listed. Complementarity determining region (CDR)3-H are the amino acid sequences of the heavy-chain in the CDR3 region. CDR3-L are the amino acid sequences of the light-chains in the CDR3 region. Total numbers of SHMs in the heavy- and light-chains are listed in the last column.
